# Catalyst-free direct vapor-phase growth of Zn_1−*x*_Cu_*x*_O micro-cross structures and their optical properties

**DOI:** 10.1186/1556-276X-8-46

**Published:** 2013-01-22

**Authors:** Danhua Xu, Donghua Fan, Wenzhong Shen

**Affiliations:** 1 Laboratory of Condensed Matter Spectroscopy and Opto-Electronic Physics, Department of Physics, Shanghai Jiao Tong University, 800 Dong Chuan Road, Shanghai, 200240, China; 2 Key Laboratory of Artificial Structures and Quantum Control (Ministry of Education), Department of Physics, Shanghai Jiao Tong University, 800 Dong Chuan Road, Shanghai, 200240, China; 3 School of Applied Physics and Materials, Wuyi University, 22 Dong Cheng Village, Jiangmen, 529020, China

**Keywords:** Cu-doped ZnO, Micro-cross structures, Optical properties, Epitaxial growth, Catalyst-free vapor-phase method

## Abstract

We report a simple catalyst-free vapor-phase method to fabricate Zn_1−*x*_Cu_*x*_O micro-cross structures. Through a series of controlled experiments by changing the location of the substrate and reaction time, we have realized the continuous evolution of product morphology from nanorods into brush-like structures and micro-cross structures at different positions, together with the epitaxial growth of branched nanorods from the central stem with the time extended. The growth mechanism of the Zn_1−*x*_Cu_*x*_O micro-cross structures has been proposed to involve the synthesis of Cu/Zn square-like core, surface oxidation, and the secondary growth of nanorod arrays. By the detailed structural analysis of the yielded Zn_1−*x*_Cu_*x*_O samples at different locations, we have shown that the CuO phases were gradually formed in Zn_1−*x*_Cu_*x*_O, which is significant to induce the usual ZnO hexagonal structures changing into four-folded symmetrical hierarchical micro-cross structures. Furthermore, the visible luminescence can be greatly enhanced by the introduction of Cu, and the observed inhomogeneous cathode luminescence in an individual micro-cross structure is caused by the different distributions of Cu.

## Background

One-dimensional (1D) ZnO nanostructures (e.g., nanowires, nanorods, and nanotubes) are promising with extensive applications in nanoelectronics and nanophotonics due to their efficient transport of electrons and excitons [1]. In recent years, increasing attention has been paid to three-dimensional (3D) hierarchical ZnO architectures which derived from 1D nanostructures as building blocks based on various novel applications [2-6]. To date, different kinds of hierarchical branched ZnO nanostructures, including nanobridges [7], nanoflowers [2,8], rotor-like structures [9], and nanotubes surrounded by well-ordered nanorod structures [10], have been reported by using either solution-phase or vapor-phase method. However, these processes often require high temperature, complex multi-step process, or introduction of impurities by the templates or foreign catalysts in the reaction system. Therefore, it is still a challenge to find a simple and controllable synthetic process to fabricate 3D hierarchical ZnO architectures with novel or potential applications.

On the other hand, doping is a widely used method to improve the electrical and optical properties of semiconductors [11]. Copper, considered as a valuable dopant for the achievement of long-searched-for p-type ZnO [12], can serve not only as a luminescence activator but also as a compensator of ZnO [13]. In addition, Cu doping, leading to form donor-acceptor complexes, can induce a polaron-type ferromagnetic order in ZnO [14,15]. Zn_1−*x*_Cu_*x*_O has been previously employed as phosphor [16], an active material in varistors [17] and spintronic devices [18]. Up to now, most of the investigations in the Zn_1−*x*_Cu_*x*_O system have been focused on thin films and 1D nanostructures, such as Cu-doped ZnO nanowires [19], nanonails, and nanoneedles [20]. 3D hierarchical Zn_1−*x*_Cu_*x*_O nanostructures, posing many unique properties arisen from their special geometrical shapes and inherently large surface-to-volume ratios, show considerable promise for the development of nanodevices with multiple functions (e.g., gas sensor [21] and photocatalytic hydrogen generation [22]). However, thus far, there have been no reports of such Zn_1−*x*_Cu_*x*_O hierarchical nanostructures.

Herein, we realize a simple catalyst-free vapor-phase deposition method to synthesize the Zn_1−*x*_Cu_*x*_O hierarchical micro-cross structures. The branched nanorods are neatly aligned on four sides of the backbone prism, assembling the shape of crosses. The subtle variations of environmental conditions have triggered the observed continuous morphological evolution from 1D nanorod to 3D hierarchical micro-cross structures. A possible growth mechanism for the micro-crosses has been proposed. Detailed structural and optical studies reveal that the CuO phases are gradually formed in Zn_1−*x*_Cu_*x*_O and Cu concentration can greatly influence the structural defects. Interestingly, the Zn_1−*x*_Cu_*x*_O micro-cross structure exhibits distinct inhomogeneous cathode luminescence (CL), which can be attributed to the different defect concentrations induced by Cu through characterizing the emission of defects and contents of Cu over the individual micro-cross structure.

## Methods

**Figure 1:**
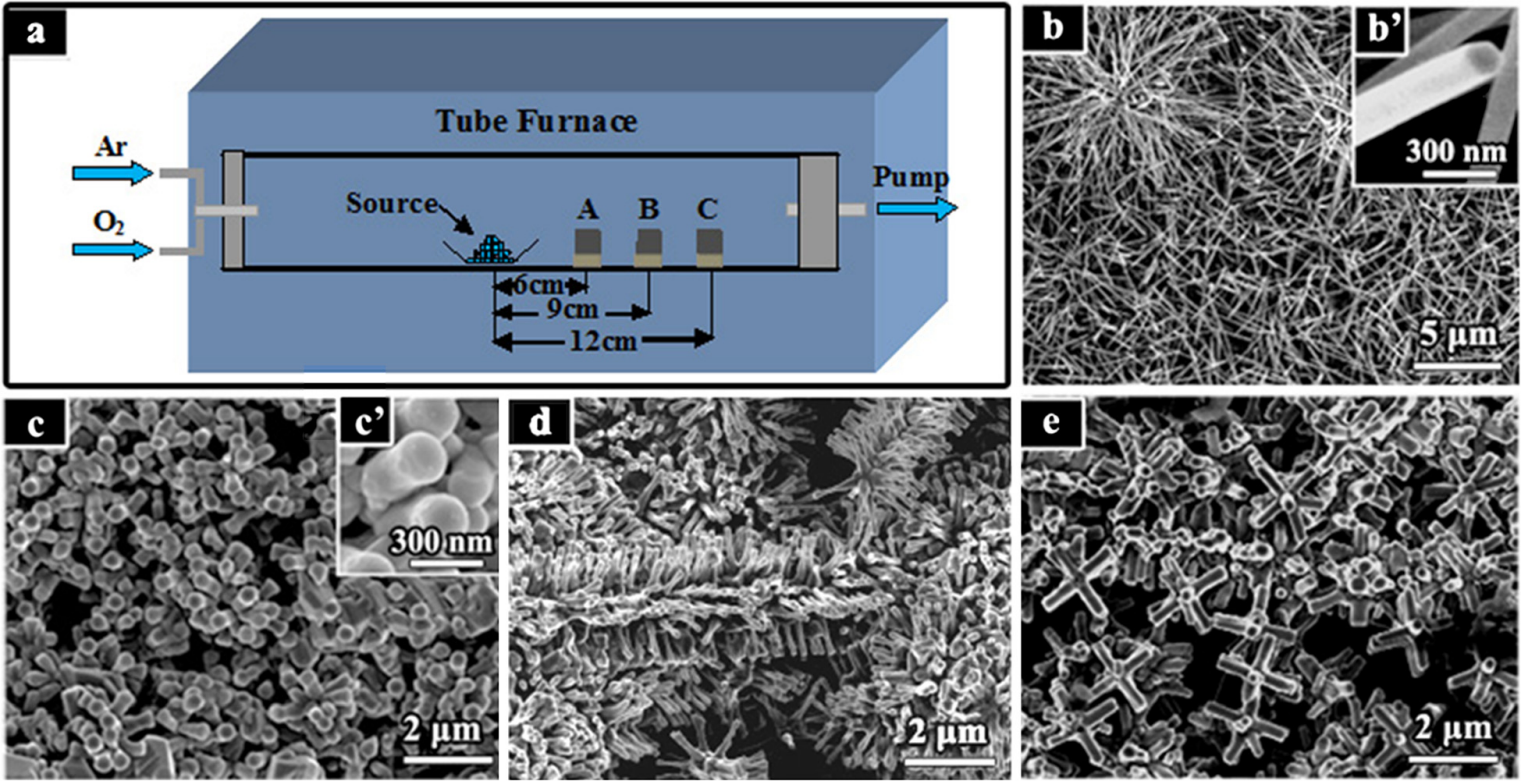
**SEM images of the as**-**fabricated samples taken at different positions.** (**a**) A schematic drawing of the experimental setup. (**b**) A FE-SEM image of pure ZnO nanowires grown without Cu in the source. (**c**, **d**, **e**) FE-SEM images of Zn_1−*x*_Cu_*x*_O samples located at positions C, B, A, respectively. Insets (**b’**) and (**c’**) show the corresponding high-magnification SEM images.

Zn_1−*x*_Cu_*x*_O nanostructures were prepared on Si substrate by a simple vapor-phase method in a horizontal tube furnace (150 cm long). Figure 1a shows the schematic drawing of the experimental setup. Zn powders (0.80 g, 99.99% purity) and Cu nanoparticle (diameter 100 to 200 nm) powders (0.32 g) were firstly mixed as the precursor substances. Due to the size effect, the copper nanoparticles can vaporize at relatively low temperatures (approximately 600°C), although the melting point of bulk copper is higher than 1,000°C. These Cu particles were synthesized by adding Zn powders into the CuCl_2_ solution via the following chemical reaction: Zn + Cu^2+^ → Zn^2+^ + Cu. The mixture was loaded into an alumina boat and placed at the center of a quartz tube (2 cm diameter, 120 cm long). N-type Si (100) wafer cleaned by sonication in ethanol and acetone was employed as the substrate and was placed about a few centimeters (from 6 to 12 cm) away from the source materials to receive the products. As we will show later, the location of the substrate appears to be an important factor determining the morphologies and the Cu contents of the final products. The quartz tube was evacuated to approximately 10 Pa using a mechanical rotary pump to remove the residual oxygen before heating. The heated temperature of the furnace was raised to 750°C at a rate of 20°C/min. When the temperature reached 350°C, argon (99.999%, 220 sccm) was introduced, and then oxygen (99.999%, 80 sccm) was added to the carrier gas at the desired temperature of 750°C. The duration of growth lasted for 5, 30, and 60 min, respectively. We finally obtained a black layer on the Si substrate after the quartz tube was cooled to room temperature naturally. For comparative studies, we have also prepared the Zn_1−*x*_Cu_*x*_O samples with different Cu contents as well as the pure ZnO nanostructure synthesized under the same experiment condition as the others but without copper source.

The morphology and microstructure of the structures were characterized by field-emission scanning electron microscopy (FE-SEM; Philips XL30FEG, Portland, OR, USA) with an accelerating voltage of 5 kV, high-resolution transmission electron microscopy (HRTEM; JEOL JEM-2100 F, Akishima-shi, Japan), and X-ray diffraction (XRD; Bruker/D8 Discover diffractometer with GADDS, Madison, WI, USA) equipped with a Cu Kα source (*λ* = 1.5406 Å). Energy-dispersive X-ray (EDX) analysis was also performed during the FE-SEM observation. The bonding characteristics were analyzed by PHI Quantum 2000 X-ray photoelectron spectroscopy (XPS; Chanhassen, MN, USA). The micro-Raman in the backscattering geometry and photoluminescence (PL) spectra were recorded at room temperature using a Jobin Yvon LabRAM HR800UV micro-Raman system (Kyoto, Japan) under Ar^+^ (514.5 nm) and He-Cd (325.0 nm) laser excitation, respectively. The CL measurements were carried out at room temperature using a Gatan Mono-CL system-attached FE-SEM (Pleasanton, CA, USA) with the accelerating voltage of 10 kV.

## Results and discussions

As a reference, specimens of pure ZnO nanostructures were grown in the tube furnace system using Zn powder as the only source material. We can observe that the as-grown products always present the commonly reported nanowire morphology (Figure 1b). The length of the undoped nanowires ranges from 4 to 8 μm, and the diameter is about 150 nm. The high-magnification SEM image is shown in Figure 1 (b’), demonstrating uniform hexagonal cross sections and a smooth surface. With the introduction of Cu in the precursor, the as-grown Zn_1−*x*_Cu_*x*_O samples exhibit three different morphologies (see in Figure 1c,d,e), which are deposited on the substrates at different positions (marked as C, B, and A in Figure 1a, respectively). For the sample at position C (as shown in Figure 1c), the nanorods are formed, of which the lengths become shorter (approximately 1.5 μm) and the diameters become bigger (approximately 250 nm). Some Zn_1−*x*_Cu_*x*_O nanorods display deformed hexagon sections (see Figure 1 (c’)), which may be induced by doping. As seen in Figure 1d, a kind of brush-like structures appears (at position B). These brushes are randomly assembled by the nanowires. For the sample at position A, the low-magnification SEM image in Figure 1e shows that a large quantity of micro-cross structures formed. The definition of micro-cross comes from the geometrical similarity to the cross structures.

**Figure 2:**
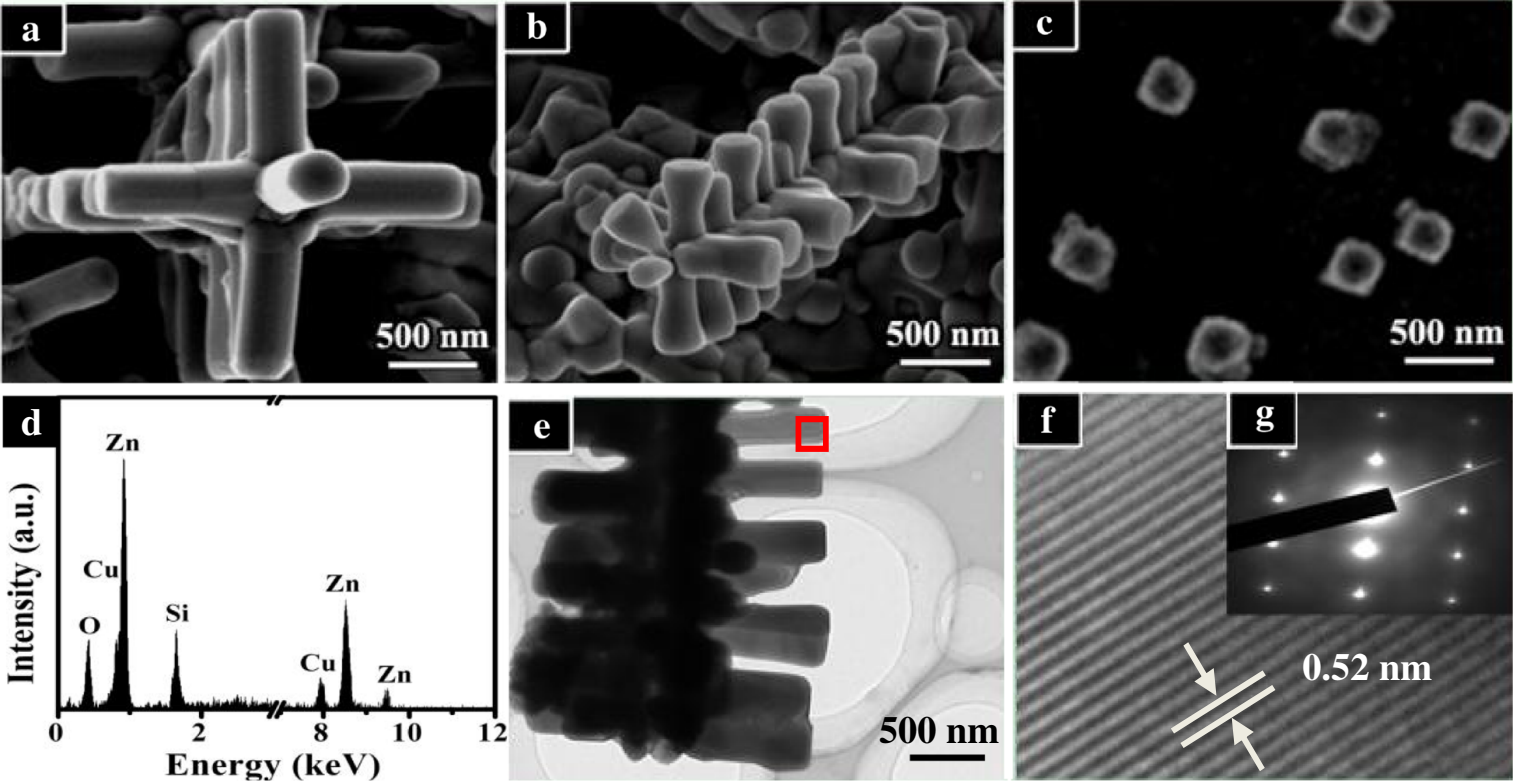
**SEM and TEM images of Zn**_**1−*x***_**Cu**_***x***_**O samples prepared for different reaction times.** (**a**, **b**, **c**) FE-SEM images of Zn_1−*x*_Cu_*x*_O nanostructures prepared at 750°C for 60, 30, and 5 min, respectively. (**d**) EDX of the sample prepared at 750°C for 5 min. (**e**) TEM image, (**f**) HRTEM image, and (**g**) SAED of Zn_1−*x*_Cu_*x*_O micro-cross structures.

Figure 2a presents the corresponding high-magnification image of such a single micro-cross. We can notice that the micro-cross is a 3D hierarchical structure, which consists of four-folded symmetrical nanorod arrays of 1 μm in length and approximately 350 nm in diameter, together with a nanorod on the central stem having a uniform hexagonal cross section. Four arrayed nanorod branches stand perpendicular to the side surfaces of the central stem. We have also reduced the reaction time to 30 and 5 min in order to observe directly the morphology evolution with the reaction time and get information about the growth process of the micro-cross structures. Under the heating time of 30 min (Figure 2b), the homogenous cross-like structures have also been formed, growing with the length of the four-folded nanorods typically reduced to approximately 450 nm. When the reaction time was 5 min, we could only obtain one-dimensional square-like nanostructures with the edge length of about 200 to 300 nm (Figure 2c), which stands for the early growth stage of the structures. The corresponding EDX analysis shown in Figure 2d indicates that the major components of the as-fabricated sample are Zn and Cu, with a small amount of oxygen.

Further morphological and structural analysis of the micro-cross structure can be characterized by the HRTEM and selected-area electron diffraction (SAED) techniques. Figure 2e presents the TEM morphology of the individual cross structure, which consists of the nanorod in the central stem, together with the nanorod arrays on the side surface of the core. The central stem is too thick to be detected from the TEM observation. The lattice fringes and the corresponding SAED pattern of the cross-like structure are shown in Figure 2f,g, respectively, which are indicated in Figure 2e with a red square. The lattice spacing of 0.52 nm corresponds to the spacing of [0001] crystal planes of wurtzite ZnO.

The above experimental observation reveals that the location of the substrate and reaction time exercise great influences on the morphologies of the products. After Cu is introduced, the ordinary pure ZnO nanowires change into three different morphologies with the variation of the location, i.e., stumpy nanorods, randomly assembled brushes, and well-organized micro-cross structures. It is speculated that the higher temperature (at position A, which is close to the central zone of the tube) is helpful to form a central core of the hierarchical structure. We could find out the clue from the original square-like core, which is shaped in the early stage of the growth process at position A (see Figure 2c). With the reaction time extended, branched nanorods grow epitaxially on the side face of the central stem (see Figure 2a,b). Since Cu has a high-symmetry cubic structure [23], we can assume that the reason for growing into four-fold hierarchical cross-like structures is because of the tetragonal-symmetry major core induced by the introduction of abundant Cu. In combination with previous reports [24,25] and the details in our experiment, we suggest the following possible growth mechanism of the Zn_1−*x*_Cu_*x*_O micro-cross structures.

At the stage of temperature rise, oxygen was still not introduced into the tube. Zn/Cu vapor easily condensed into a square-like core on the substrate. When the temperature reached up to the desired 750°C, the core was oxidized with the introduction of oxygen. The cubic core prism could provide its four prismatic facets as growth platforms for the secondary branched nanorod arrays. With the successive arrival of Zn/Cu and O_2_, the branched nanorods began to grow perpendicular to the central stem. Due to the considerable anisotropy in the speed of the crystal growth along different directions of ZnO, the nanorods with the right orientation, i.e., with the [0001] direction perpendicular to the surface of the prism, could grow much faster than others. The lengths of the branched nanorods are increased with the growth time extended (see Figure 2a,b). In the whole growth process, there are no external metallic catalysts (e.g., Au and In) involved in the formation of micro-cross structures. That is, the 3D hierarchical micro-cross structure is synthesized by a simple catalyst-free direct vapor-phase growth method.

**Figure 3:**
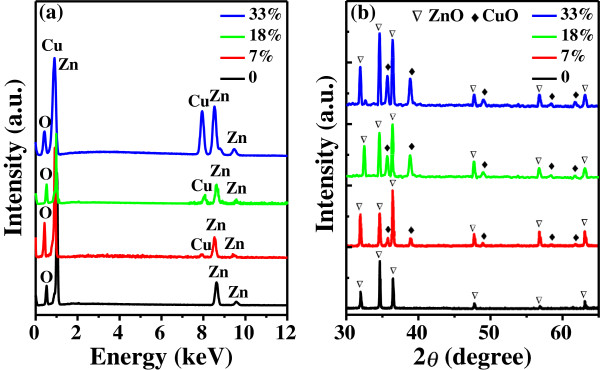
**EDX and XRD spectra.** (**a**) EDX and (**b**) XRD spectra of undoped ZnO and Zn_1−*x*_Cu_*x*_O samples with the Cu content of 7%, 18%, and 33%.

Figure 3a presents the corresponding EDX spectra of the yielded samples at different locations, which exhibit different Cu concentrations. The undoped ZnO nanostructures (noted as ‘0’ for ZnO) is used as a reference. Its EDX analysis indicates that the obtained structures are composed of only Zn and O elements. After adding Cu powder in the precursor, the appearance of the element Cu demonstrates that Cu is introduced successfully in the as-fabricated samples. From the atomic ratio of Cu to Zn in the EDX spectra, we can determine the molar ratio of Cu to (Cu + Zn) in the Zn_1−*x*_Cu_*x*_O samples (from positions A to C in Figure 1a) to be *x* = 0.33, 0.18, and 0.07, respectively. The Cu vapor is more easily condensed on the substrate at the position closer to the central zone.

The structural phase evolution of the as-fabricated products with different Cu concentrations was also investigated by XRD, which is shown in Figure 3b. It is clear that all the diffraction peaks can be indexed to the hexagonal wurtzite structure of ZnO (JCPDS No. 36–1451) in the undoped one. In contrast, five small new phases emerge in the sample with the Cu content of 7%. These new phases in the XRD spectrum correspond to CuO (matched with JCPDS No. 01–1117), owing to the fact that the solubility of Cu ions in ZnO is quite low [12]. Moreover, it is noted that with the increase of Cu content, these CuO diffraction peaks become more obvious and stronger. Meanwhile, the ZnO diffraction peaks remain nearly unshifted, indicating that the added Cu elements have no effects on the crystal structure of ZnO, which is coincident with the HRTEM results in Figure 2f.

**Figure 4:**
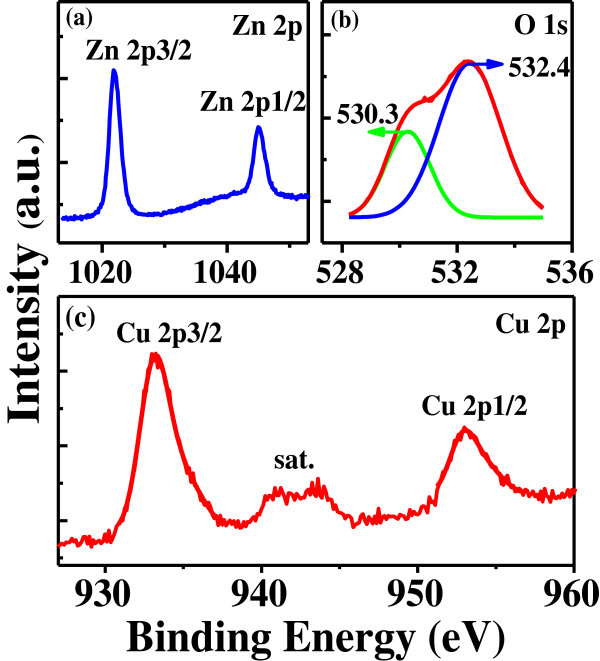
**XPS spectra.** High-resolution XPS spectra of (**a**) Zn 2*p*, (**b**) O 1*s*, and (**c**) Cu 2*p* in micro-cross structures of Zn_0.67_Cu_0.33_O.

Further evidence for the component of the as-prepared samples is obtained by XPS measurement, which is an excellent technique for understanding the oxidation state of the copper ion in ZnO. Figure 4 illustrates the high-resolution XPS spectra of Zn 2*p*, O 1*s*, and Cu 2*p* in the sample with the highest Cu content of 33% (a typical concentration in this work). As shown in Figure 4a, the XPS spectrum of Zn 2*p* reveals the binding energies of Zn 2*p*_3/2_ at about 1,021.8 eV and Zn 2*p*_1/2_ centered at 1,045.1eV, without any noticeable shift after the high-Cu doping [26]. The XPS spectrum of O 1*s* (Figure 4b) is broad and asymmetric, indicating the presence of multi-component oxygen species. It can be resolved by using a curve fitting procedure: one is located at 530.3 eV and the other one is located at 532.4 eV. The former is inherent O atoms bound to metals (such as Cu and Zn), while the latter is associated with adsorbed oxygen [27]. Figure 4c shows the core-level and shake-up satellite (sat.) lines of Cu 2*p*. The Cu 2*p*_3/2_ and 2*p*_1/2_ core levels are located at *ca.* 933.2 and *ca.* 952.9 eV, respectively, which are close to the data for Cu 2*p* in CuO [28]. In our samples, it is easy to observe two shake-up satellites at about 8.7 and 10.9 eV above the main 2*p*_3/2_ peak. The existence of strong satellite features for Cu 2*p* rules out the possibility of the presence of Cu_2_O phase [29], corresponding well with the XRD observation in Figure 3b.

**Figure 5:**
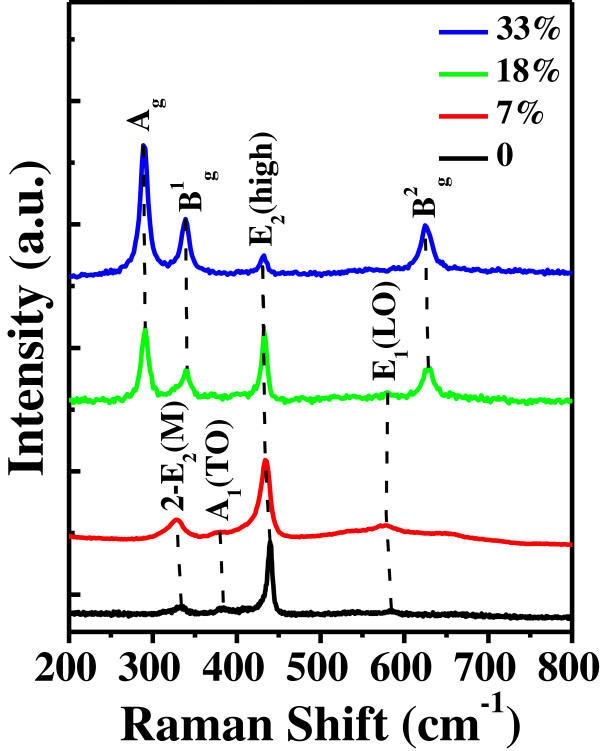
**Raman spectra.** Raman spectra of undoped ZnO and Zn_1−*x*_Cu_*x*_O samples with the Cu contents of 7%, 18%, and 33%.

Figure 5 shows the Raman spectra of both the undoped ZnO and Zn_1−*x*_Cu_*x*_O nanostructures with different Cu contents in the range 200 to 800 cm^−1^ measured at room temperature. In the undoped ZnO sample, the peaks at 331, 384, and 584 cm^−1^ correspond to the second-order acoustic (2-E_2_(M)) mode, A_1_ transverse optical (A_1_(TO)) mode, and E_1_ longitudinal optical (E_1_(LO)) mode, respectively [30]. The sharp and strong peak at around 437 cm^−1^ can be attributed to the high-frequency branch of the E_2_ (E_2_(high)) mode of ZnO, which is the strongest, and typical Raman-active branch of the wurtzite crystal structure [31]. In the Zn_1−*x*_Cu_*x*_O nanostructures, the presence of the E_2_(high) mode confirms that they all have a typical hexagonal wurtzite structure, which is consistent with the above HRTEM and XRD observations. When the Cu content is 7%, the E_2_(high) and E_1_(LO) modes become broader and shift to lower frequency, as compared with the undoped counterpart. This may be due to the decrease in the binding energies of Zn-O bonds as a result of the Cu doping, indicating that the long-range order of the ZnO crystal is destroyed by Cu dopants [32].

On the other hand, three additional modes at around 290, 340, and 628 cm^−1^ can be observed. They are attributed to the A_g_, B^1^_g_, and B^2^_g_ modes of CuO due to the vibrations of oxygen atoms, respectively [33,34]. From Figure 5, it is obvious that the intensity of the CuO peaks enhanced while that of ZnO peaks decreases with the Cu concentration increases up to 33%. Such behavior is caused by the competition of Zn and Cu during the oxidization process. In the sample with the highest Cu content of 33%, the formation of CuO is dominant, in spite of the fact that the lower melting point and higher vapor pressure of Zn than those of Cu under the same conditions [35]. The formation of CuO is significant to induce the usual ZnO hexagonal structures changing into four-folded cross-like structures, in good agreement with the growth mechanism we have proposed above.

**Figure 6:**
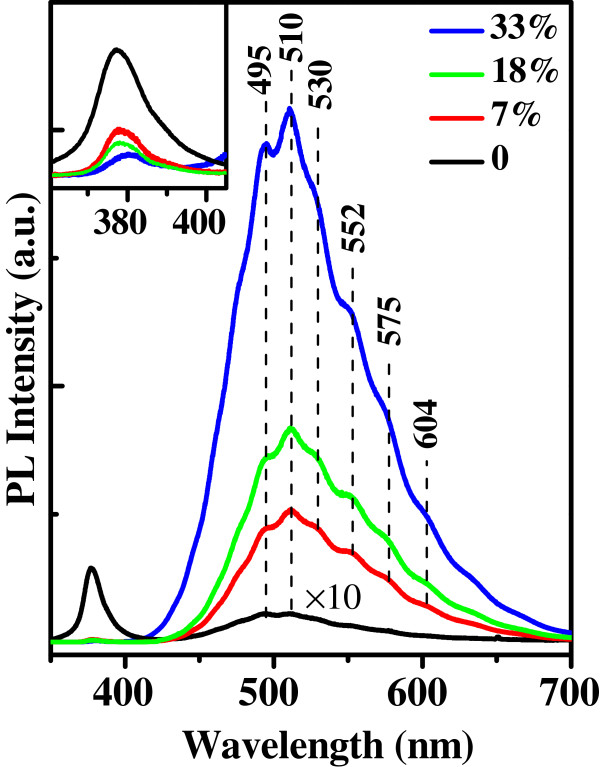
**PL spectra.** PL spectra of undoped ZnO and Zn_1−*x*_Cu_*x*_O samples with the Cu contents of 7%, 18%, and 33%.

In order to investigate the effects of the different Cu concentrations on the optical characteristics in the yielded samples, we have carried out PL spectroscopy as shown in Figure 6. We can see that all the samples show two emission peaks: a sharp one appearing at approximately 377 nm in the ultraviolet (UV) region and another broad one in the visible region. The former is ascribed to the near-band-edge (NBE) exciton recombination, while the latter is quite complicated due to the native and dopant-induced defects in ZnO. The intensive PL emission peak at 495 nm is suggested to be mainly due to the presence of various point defects, which can easily form recombination centers. The peak corresponding to 510 nm is usually generated by the recombination of electrons in singly ionized oxygen vacancies with photogenerated holes in the valence band [36,37]. Apart from the strong peaks at 495 and 510 nm, the visible band consists of at least four sub-peaks at wavelengths of 530, 552, 575, and 604 nm, resulting from the local levels in the bandgap of ZnO. The green shoulders at 530 and 552 nm are attributed to the antisite oxygen and interstitial oxygen, respectively [35]. The peak at 604 nm is possibly caused by the univalent vacancies of zinc in ZnO. The origin of another peak at 575 nm has been rarely mentioned and is still unclear.

As can be clearly observed from Figure 6, the undoped ZnO possesses a strong near-band-edge UV emission together with a weak visible emission, indicating that the undoped ZnO nanostructures have a fairly high quality with low defect concentration (its PL intensity was 10 times magnified). After Cu is introduced, the UV emission is rapidly suppressed while the visible luminescence is greatly enhanced compared with the undoped counterpart, suggesting the poorer crystallinity and greater level of structural defects introduced by Cu ion incorporation into ZnO. The intensity ratio of the visible band emission to the UV peak increases from approximately 0.2 to approximately 150 with the Cu content change from 0% to 33%, demonstrating that the Cu doping strongly increases the concentration of defects. Nevertheless, the defects are believed to significantly improve a variety of surface properties, such as heterogeneous catalysis, corrosion inhibition, and gas sensing, which have been addressed by theoretical calculation and experimental data [38-40]. Furthermore, we have also presented in the inset the enlarged view of the UV peak between 360 and 405 nm. It is obvious that the introduction of Cu will cause a little redshift of the UV peak (34 meV under Cu contents from 0% to 33%) compared with the undoped one, i.e., a reduction of ZnO bandgap caused by the Cu doping.

**Figure 7:**
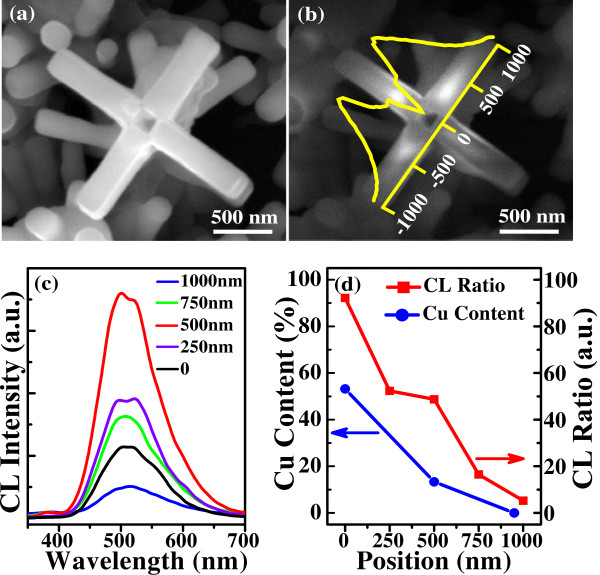
**SE and CL images of a single micro**-**cross structure with its corresponding spectra.** (**a**) SE image of the Zn_1−*x*_Cu_*x*_O micro-cross. (**b**) CL panchromatic image padded with the brightness distribution curve along the axial line of the sample. (**c**) Corresponding CL spectra at five different locations along the axial line of one branched nanorod. (**d**) CL ratio and Cu content variation with different positions of the branched nanorod.

We have also employed the high-spatial resolution CL technique at various locations within the same cross structure to explore the defect distribution and the local optical properties in an individual Zn_1−*x*_Cu_*x*_O micro-cross. A typical secondary electron (SE) image of such an individual micro-cross is shown in Figure 7a. Clearly, there is a 200-nm square hole in the center of the stem, which confirms that the central zone is a cubic prism. Figure 7b presents the corresponding panchromatic CL image at the same place. Interestingly, the cross structure exhibits inhomogeneous luminescence. The strong CL emissions are mainly focused on the middle of the four-folded branched nanorod according to the intense distribution curve obtained along the axial line (yellow curve).

Figure 7c illustrates the typical CL spectra, which are acquired at the center stem (noted as ‘0’ on the axis in Figure 7b) and four different locations along one branched nanorod. The spectra exhibit similar features as the PL spectra, that is, a comparatively weak UV peak due to the NBE emission and a broad, strong peak in the visible region, which is attributed to the deep-level (DL) emission affiliated with defects and impurities. To further reveal the variation of the defect concentration, the intensity ratios of the DL emission to the NBE emission (*I*_DL_/*I*_NBE_) at different locations are plotted in Figure 7d (marked as ‘CL Ratio’). We can notice that the ratio of *I*_DL_/*I*_NBE_ decreases from approximately 92 to approximately 5 with the location change from 0 to 1,000 nm, demonstrating that the concentration of defects strongly depends on the location. The center part of the cross-like structure exhibits the highest defect density. We have also performed the EDX analysis on three different location points along the branched nanorod to illustrate the evolution of the Cu content (marked as ‘Cu Content’ in Figure 7d). It is clear that the central zone of the cross structure has the higher Cu concentration of approximately 53.6%, while the edge part of the branched nanorod has ultra-low Cu content (nearly zero). The introduction of abundant Cu in the core has induced the usual ZnO hexagonal structures changing into four-folded symmetrical micro-cross structures, which is consistent with the abovementioned growth mechanism and EDX analysis (shown in Figure 2d). The Cu contents are consistently and significantly reduced from the central zone to the edge part of the branched nanorod, which may be caused by the Cu diffusion at the stage of epitaxial growth of branched nanorods from the central core. The spatial differences of the Cu content along the structure would induce the variation of the defect distribution, resulting in the distinct inhomogeneous luminescence within one micro-cross structure.

## Conclusions

In summary, we report a new and delicate cross-like Zn_1−*x*_Cu_*x*_O structure, in which four-sided branched nanorod arrays grow perpendicular to the side surfaces of the central stem. This structure is formed through the direct vapor-phase deposition method but without introducing any catalyst. By changing the reaction time, the possible growth mechanism of the micro-cross structures has been proposed to involve the synthesis of Cu/Zn core, surface oxidation, and the secondary growth of the branched nanorods. The location of the substrate is an important factor determining the morphologies (from 1D nanorods to 3D micro-cross structures) and Cu concentrations (from 7% to 33%) of the yielded Zn_1−*x*_Cu_*x*_O samples. We have employed the XRD, Raman, and PL spectroscopies to demonstrate that the formation of CuO-related phases and concentration of the defects in the products have been greatly influenced by the Cu content. Moreover, inhomogeneous CL has been observed in a single micro-cross structure, which is generated from structural defects created by the Cu incorporation into ZnO. The presented method is expected to be employed in a broad range to fabricate other similar metal-doped ZnO 3D hierarchical structures for their potential device applications.

## Abbreviations

1D: One-dimensional; 2-E_2_(M): Second-order acoustic; 3D: Three-dimensional; A_1_(TO): A_1_ transverse optical; CL: Cathode luminescence; DL: Deep level; E_1_(LO): E_1_ longitudinal optical; E_2_(high): High-frequency branch of the E_2_; EDX: Energy-dispersive X-ray; FE-SEM: Field-emission scanning electron microscopy; HRTEM: High-resolution transmission electron microscopy; NBE: Near-band-edge; PL: Photoluminescence; SAED: Selected-area electron diffraction; SE: Secondary electron; UV: Ultraviolet; XPS: X-ray photoelectron spectroscopy; XRD: X-ray diffraction.

## Competing interests

The authors declare that they have no competing interests.

## Authors’ contributions

DHX participated in the design of the study, carried out the experiments, and performed the statistical analysis, as well as drafted the manuscript. DHF participated in the design of the study and provided the experimental guidance. WZS took charge of the theoretical guidance and revised the manuscript. All authors read and approved the final manuscript.
